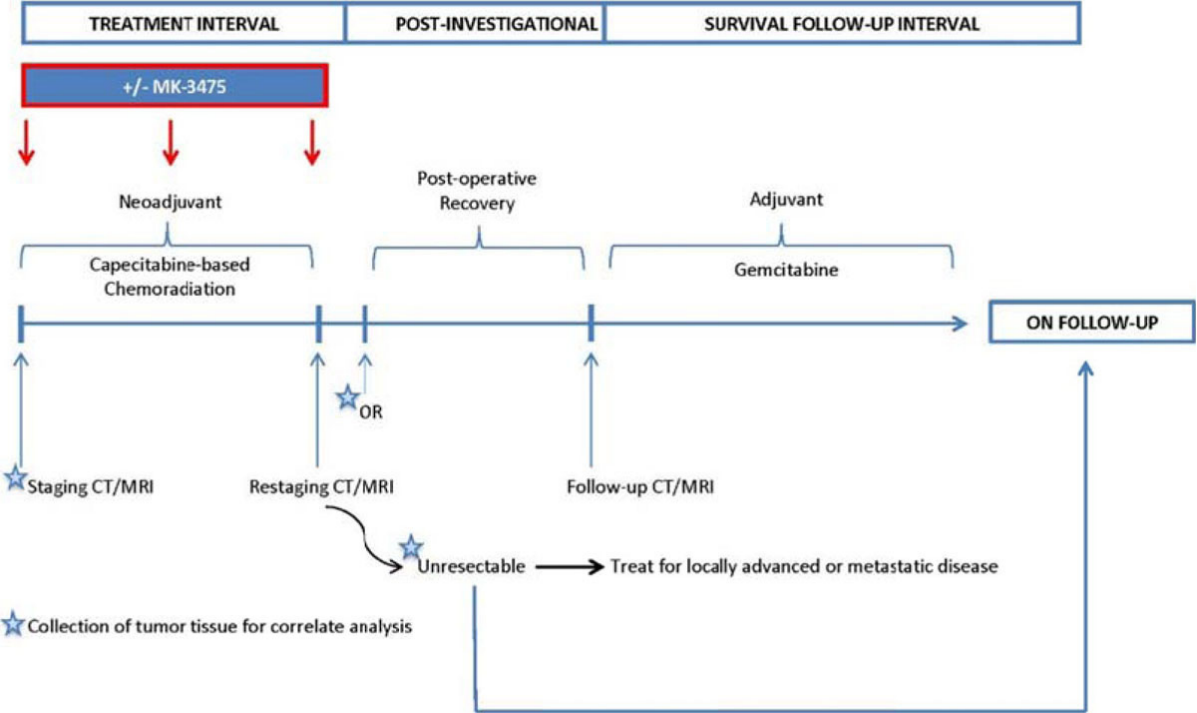# A randomized multicenter Phase Ib/II study to assess the immunological effect of chemoradiation therapy (CRT) in combination with pembrolizumab compared to CRT alone in resectable or borderline resectable pancreatic cancer

**DOI:** 10.1186/2051-1426-3-S2-P167

**Published:** 2015-11-04

**Authors:** Matthew Katz, Bauer W Todd, Gauri Varadhachary, Nicolas Acquavella, Gina Petroni, Timothy Bullock, Craig L Slingluff, Osama Rahma

**Affiliations:** 1 MD Anderson, Houston, TX, USA; 2 UVA, Charlottesville, VA, USA; 3 University of Miami, Miami, FL, USA; 4 University of Virginia, Charlottesville, VA, USA

## Background

Tumor-infiltrating lymphocytes (TILs) play a major role in anti-tumor immune responses, and their presence is correlated with survival in a variety of tumors. These TILs do not reach the pancreatic cancer (PC) cells in significant numbers due to the presence of stroma and a suppressive microenvironment. Neoadjuvant chemoradiation therapy (CRT) has been advocated as a potential way to improve outcomes of patients with resectable or borderline resectable PC. More importantly, there is recent evidence to suggest that CRT can increase the presence of TILs in the PC microenvironment (PCME), leading to production of interferon-γ (IFN-γ), which could increase the expression of PD-L1 through a negative feedback loop. Accordingly, we hypothesize that blocking the PD-1 receptor will synergize with CRT to increase the density and activation of TILs in the PCME.

## Methods

This is a prospective multicenter randomized trial which will accrue 45 subjects with resectable or borderline resectable pancreatic cancer who had not received prior treatment for PC and have ECOG of 0-1. The primary objectives of the study are: (1) to determine the safety of neoadjuvant CRT in combination with pembrolizumab. (2) To estimate the difference in the number of TILs in pancreatic cancer subjects receiving neoadjuvant CRT in combination with pembrolizumab to the number of TILs in subjects receiving neoadjuvant CRT alone. Eligible subjects will be randomized 2:1 to the investigational treatment (Arm A) to receive pembrolizumab administered IV every 3 weeks on days 1, 22, and 43 during concurrent CRT with capecitabine (825 mg/m2 orally twice daily, Monday (M) through Friday (F), on days of radiation only) and radiation (50.4 Gy in 28 fractions over 28 days) or Arm B to receive only concurrent CRT with capecitabine. In all subjects, restaging CT scan or MRI will be performed at 4-6 weeks after completion of neoadjuvant treatment to determine resectability. Patients without local or distant disease progression will be taken to the operative room for planned surgery (within 2 weeks of imaging). Postoperatively, resected patients will receive off study standard of care adjuvant gemcitabine (1000mg/kg IV weekly for 3 out of 4 weeks for 6 months). Post operatively resected patients will be followed for up for PFS and OS for up to 2 years. Participants are considered evaluable if they satisfy all inclusion and exclusion criteria and have tumor tissue samples sufficient to determine the number of TILs. Clinical Trial Registry Number (NCT02305186).

## Trial registration

ClinicalTrials.gov identifier NCT02305186.

**Figure 1:**
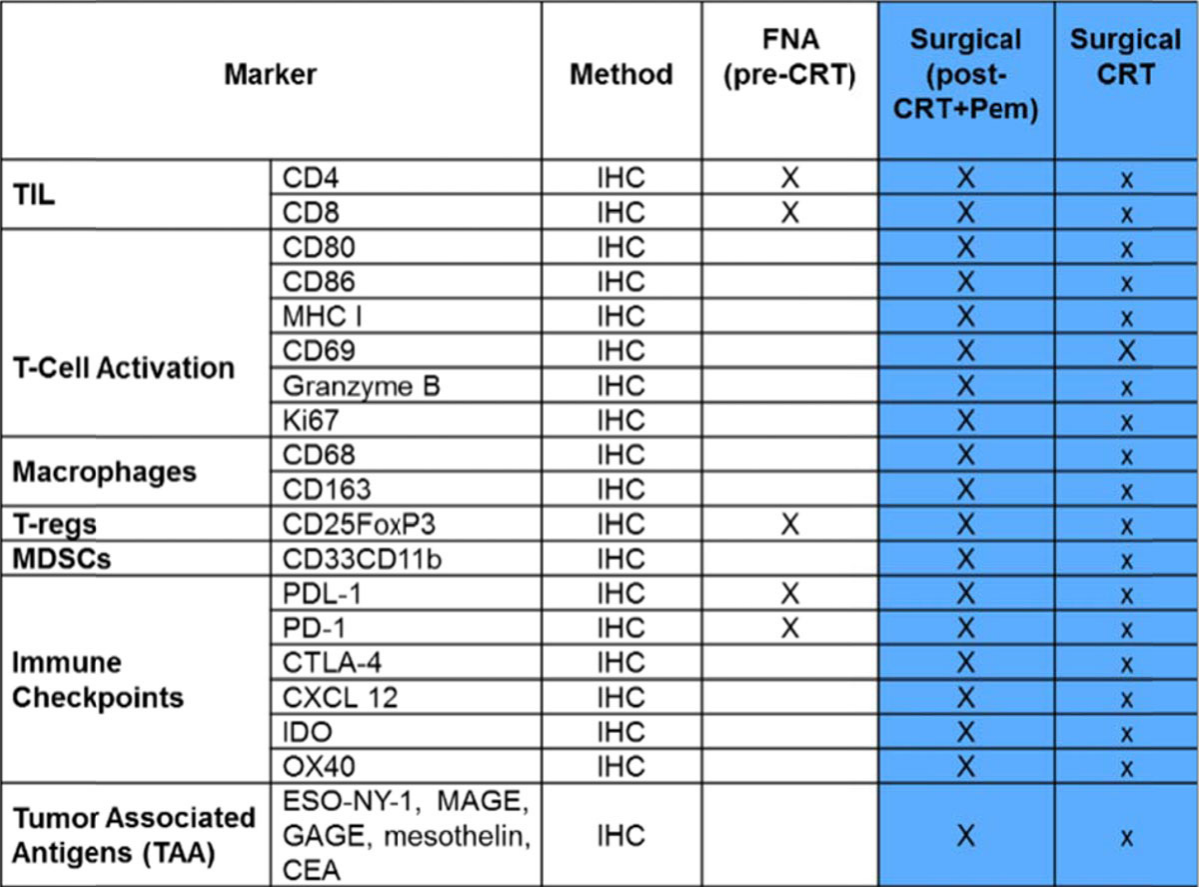


**Figure 2:**